# Integrated Analysis of the Roles of Long Noncoding RNA and Coding RNA Expression in Sheep (*Ovis aries*) Skin during Initiation of Secondary Hair Follicle

**DOI:** 10.1371/journal.pone.0156890

**Published:** 2016-06-08

**Authors:** Yaojing Yue, Tingting Guo, Chao Yuan, Jianbin Liu, Jian Guo, Ruilin Feng, Chune Niu, Xiaoping Sun, Bohui Yang

**Affiliations:** Lanzhou Institute of Husbandry and Pharmaceutical Sciences, Chinese Academy of Agricultural Sciences, Jiangouyan Street, Lanzhou, China; University of California, Davis, UNITED STATES

## Abstract

Initiation of hair follicle (HF) is the first and most important stage of HF morphogenesis. However the precise molecular mechanism of initiation of hair follicle remains elusive. Meanwhile, in previous study, the more attentions had been paid to the function of genes, while the roles of non-coding RNAs (such as long noncoding RNA and microRNA) had not been described. Therefore, the roles of long noncoding RNA(LncRNA) and coding RNA in sheep skin during the initiation of sheep secondary HF were integrated and analyzed, by using strand-specific RNA sequencing (ssRNA-seq).A total of 192 significant differentially expressed genes were detected, including 67 up-regulated genes and 125 down-regulated genes between stage 0 and stage 1 of HF morphogenesis during HF initiation. Only *Wnt2*, *FGF20* were just significant differentially expressed among Wnt, Shh, Notch and BMP signaling pathways. Further expression profile analysis of lncRNAs showed that 884 novel lncRNAs were discovered in sheep skin expression profiles. A total of 15 lncRNAs with significant differential expression were detected, 6 up-regulated and 9 down-regulated. Among of differentially expressed genes and LncRNA, XLOC002437 lncRNA and potential target gene *COL6A6* were all significantly down-regulated in stage 1. Furthermore, by using RNAhybrid, XLOC005698 may be as a competing endogenous RNA ‘‘sponges” oar-miR-3955-5p activity. Gene Ontology and KEGG pathway analyses indicated that the significantly enriched pathway was peroxisome proliferator-activated receptors (PPARs) pathway (corrected P-value < 0.05), indicating that PPAR pathway is likely to play significant roles during the initiation of secondary HF.Results suggest that the key differentially expressed genes and LncRNAs may be considered as potential candidate genes for further study on the molecular mechanisms of HF initiation, as well as supplying some potential values for understanding human hair disorders.

## Introduction

Hair follicle (HF) research is a rapidly developing area of skin biology [1]. HF morphogenesis and cycling can be successfully used for a wide range of studies into the mechanisms of morphogenesis [2], stem cell behavior [3], cell differentiation [2] and apoptosis [1, 4]. Moreover, HF investigations can provide invaluable insights into the possible causes of human hair disorders [5–8].

The morphogenesis of HF is an excellent example of mesenchymal-epithelial interactions [9]. HF formation has been divided into eight distinct developmental stages (0–8). The stages of morphogenesis are broadly classified as follows: induction (stages 0–1), organogenesis (stages 2–5) and cytodifferentiation (stages 6–8) [10]. The first stage, stage 0 of HF morphogenesis, corresponds to the undifferentiated and single-layered epidermis with no morphological signs of HF induction. Classically, the initiation of HF is described in terms of an ordered series of mesenchymal-epithelial interactions. At stage 1(also described as hair placode stage), a “first dermal message” emanating from the dermis acts on an unspecified epidermis, and the formation of morphologically recognizable hair placodes occurs [11]. In sheep embryos, second HF placodes are formed at E96 [12]. These placodes then emit an epidermal signal that instructs underlying mesenchymal cells to cluster and form “dermal condensates.” A “second dermal message” from the dermal condensates induces epidermal placode cells to rapidly divide downward and invade the dermis, thus enwrapping the dermal condensate, which becomes the HF germ (stage 2) [13]. Although the precise nature of the epidermal placode-inducing “first dermal message” remains poorly understood, several studies have suggested that HF initiation is an orchestrated interaction between mesenchymal and epithelial cells mediated through the secretion of stimulator and inhibitor signaling molecules, such as *Wnt*/*β-catenin*, *EDA*/*EDAR*/*NF-κB*, *Noggin*/*Lef-1*, *Shh*, *BMP-2*/*4*/*7* and *FGF* [9, 14]. Recent studies looking beyond protein-coding genes have shown that non-coding RNA (ncRNA), such as microRNA(miRNA),natural antisense transcripts (NAT) and long non-coding RNA (lncRNA), can show higher specificity as biomarkers for some applications than protein coding genes [15–19]. Among these ncRNAs, lncRNAs are not only large in quantity but also play important roles in gene expression regulation in organisms [20–22]. However, few lncRNAs are annotated within the sheep genome. LncRNAs are generally defined as having a size greater than 200 nucleotides, and they constitute a diverse group of non-coding RNAs that are distinct from miRNAs [23]. LncRNAs have been implicated in biological, developmental and pathological processes, and they act through mechanisms such as chromatin reprogramming, *cis* regulation at enhancers and post-transcriptional regulation of mRNA processing [15, 21, 22, 24]. However, the roles of lncRNAs in controlling HF initiation have not been described. In this study, we used strand-specific RNA sequencing (ssRNA-seq) to identify the role of lncRNAs and mRNAs in sheep skin during the initiation of secondary HF.

## Results

### Sequencing and assembly

**Fig 1:**
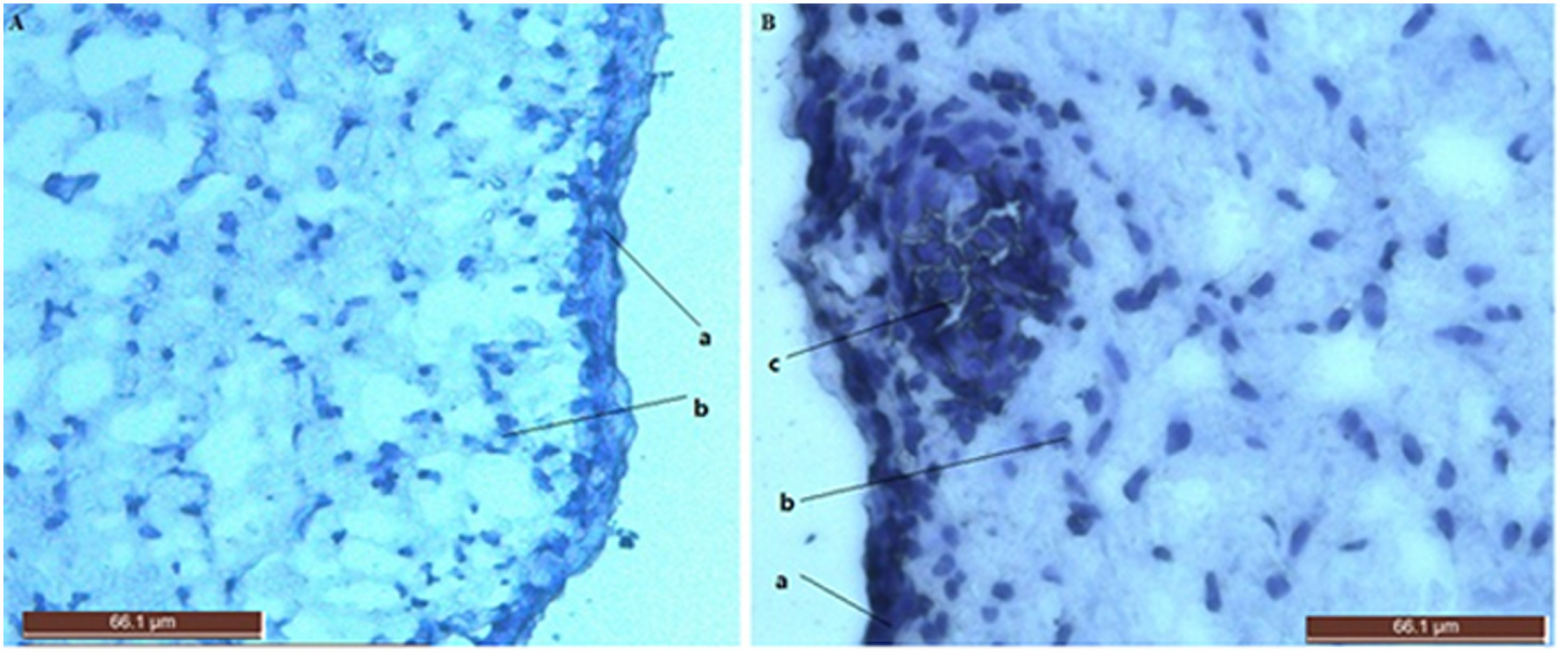
Skin sections during the initiation of secondary hair follicle. a.Stage 0 of secondary HF morphogenesis in 87E fetal ovine skin sections(200×).hematoxylin stained section. (a)Epidermis; (b)eukaryotic cells.b.Stage 1 of secondary HF morphogenesis in 96 E fetal ovine skin sections(400×). (a)Epidermis; (b)eukaryotic cells;(c)hair placode.

NGS was performed on two groups, stage 0 of HF morphogenesis (n = 3) (Fig 1A) and stage 1 of HF morphogenesis (n = 3) (Fig 1B), and raw reads greater than 100 million were obtained for every group (Table 1). There was a 3' adaptor sequence in the raw data as well as small amounts of low-quality sequences and various impurities. Impurity data were removed from the raw data. For the stage 0 and stage 1 libraries, 103,413,896 and 95,671,374 clean reads were obtained, respectively.

**Table 1:** Summary of clean reads mapping to the Ovis_aries_v3.1 reference genome sequence.

Sample name	Stage 0	Stage 1
Total reads	103413896	95671374
Total mapped	87319755 (84.44%)	80291861 (83.92%)
Multiple mapped	4739091 (4.58%)	3933783 (4.11%)
Uniquely mapped	82580664 (79.85%)	76358078 (79.81%)
Reads map to '+'	41274699 (39.91%)	38168756 (39.9%)
Reads map to '-'	41305965 (39.94%)	38189322 (39.92%)

The reads were then aligned using Top Hat [25] onto the Ovis_aries_v3.1 reference genome sequence. For the stage 0 and stage 1, 84.44% and 83.92% of the reads were aligned with the reference sheep genome, respectively (Table 1), and 79.85% and 79.81% of the clean reads were uniquely located in the reference sheep genome, respectively. Moreover, 41,274,699 (39.91%) and 38,168,756 (39.9%) clean reads of the stage 0 and stage 1 groups, respectively, were mapped to the positive strand of the reference sheep genome. Furthermore, 41,305,965 (39.94%) and 38,189,322 (39.92%) clean reads of the stage 0 and stage 1 libraries, respectively, were mapped to the negative strand of the reference sheep genome.

**Fig 2:**
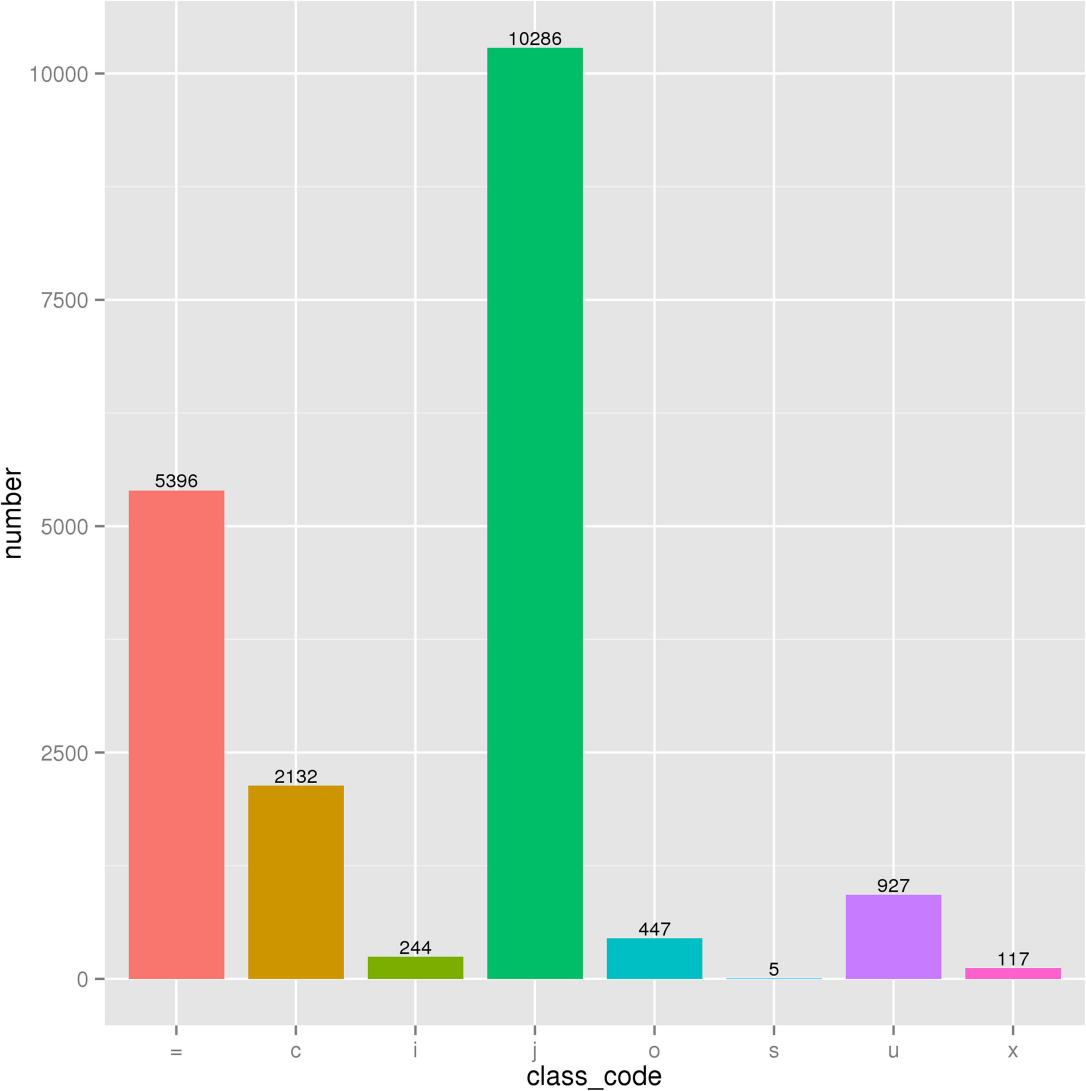
Distribution of sheep skin transcripts by Cufflinks class code. =, Complete match of intron chain; c, Contained by a reference transcript;I, A transfrag falling entirely within a reference intron; j, At least one splice junction is shared with a reference transcript; o, Generic exonic overlap with a reference transcript; s, An intron of the transfrag overlaps a reference intron on the opposite strand; u, Unknown, intergenic transcript; x, Exonic overlap with reference on the opposite strand.

Out of the annotated transcripts of Stage 0 and Stage 1, 22,572,607 (61.92%) and 20,955,431 (62.51%) transcripts, respectively, were identified as protein-coding mRNAs, while 1.67% and 2.41%, respectively, were classified as different types of noncoding transcripts, such as miscRNA, pseudo gene, rRNA and tRNA. The remaining other types of transcripts amounted to 13,273,937 (36.41%) and 11,759,533 (35.08%) for stage 0 and stage 1, respectively, and these transcripts may include lncRNAs (Fig 2).

### Identification of lncRNAs

**Fig 3:**
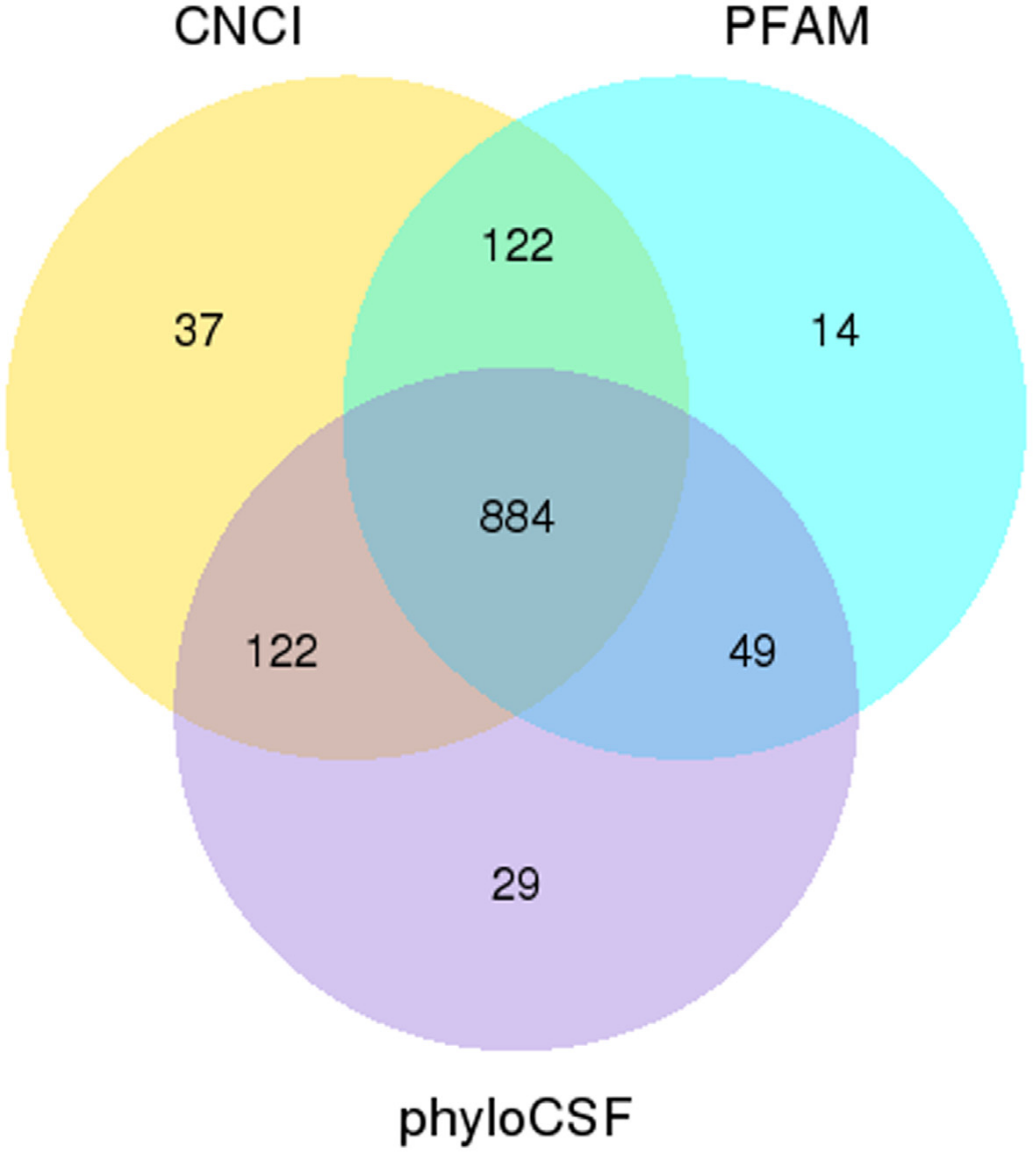
Venn diagram of the number of LncRNA with coding potential analysis by CNCI, pfam and phyloCSF.

After sequence assembly with Cufflinks [26] and scripture [27], the similar or identical transcripts to the known sheep non-mRNA (rRNA, tRNA, snRNA, snoRNA, pre-miRNA and pseudogenes) were filtered out from 20,142 transcripts matching both stitching software using Cuffcompare, and the obtained transcripts were then compared with the known mRNA of the reference sheep genome. The class_code information in the results of Cuffcompare (http://cole-trapnell-lab.github.io/cufflinks/) was used to screen the candidate transcripts. The transcripts annotated by "i", "u" and "x" from class_code were used as the candidate lncRNA for lincRNA, intronic lncRNA and anti-sense lncRNA, respectively, resulting in a total of 1288 lncRNA candidate transcripts (Fig 2). The candidate lncRNAs with coding potential were excluded using Coding-Non-Coding Index (CNCI), PhyloCSF and pfam protein domain analysis, which resulted in 884 lncRNAs (Fig 3) including 622 lincRNAs, 188 intronic lncRNAs and 74 anti-sense lncRNAs. Analysis of the characteristics of the sheep lncRNA and the transcripts encoding a protein showed that sheep lncRNAs were significantly shorter than the mRNAs in length distribution (S1A Fig) and ORF length (S1B Fig). Moreover, the number of exons was also less than that of mRNAs (S1C Fig), and the sheep lncRNAs were longer than human and mouse lncRNAs [28]. The sequence conservation of mRNA and lncRNA were conservatively scored using phastCons, resulting in the cumulative distribution curve of mRNA and LncRNA conservation scores (S1D Fig), which indicated that the sequence conservation of lncRNA was less than that of mRNA.

### Differentially expressed genes and lncRNAs

**Fig 4:**
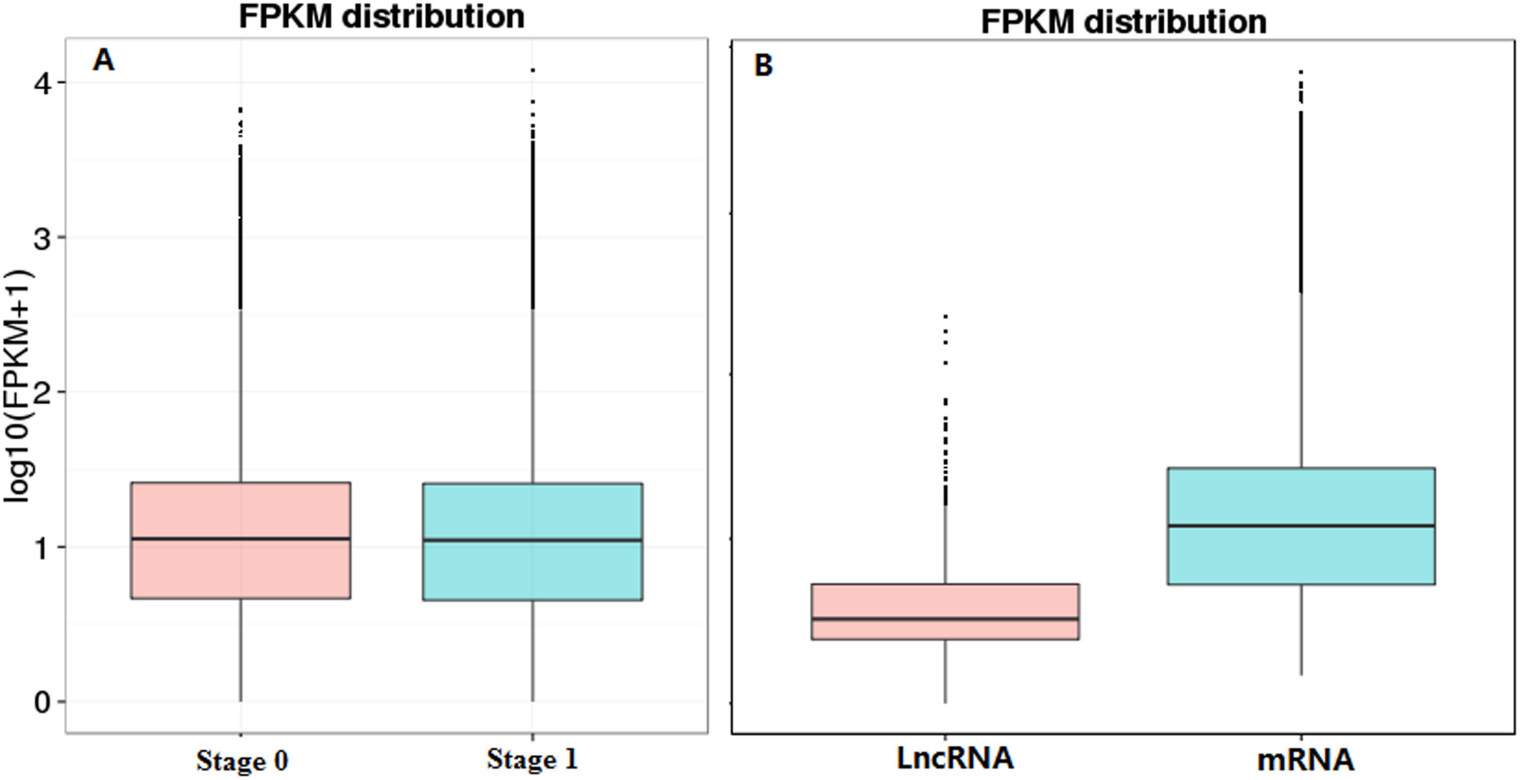
The FPKM distribution of mRNA and lncRNA expression in sheep skin during the initiation of secondary HF. (a) The FPKM density distribution of tanscript expression in sheep skin at stage 0 and stage 1.(b) the expression level of mRNA and lncRNA in sheep skin during the initiation of secondary HF.

The mRNA and lncRNA expression was analyzed using Cuffdiff in Cufflinks [28] software. In sheep skin during the initiation of secondary HF, the gene expression level was low, and the gene expression levels of the two groups were similar (Fig 4a). However, the expression level of mRNA was higher than that of lncRNA (Fig 4b).

**Fig 5:**
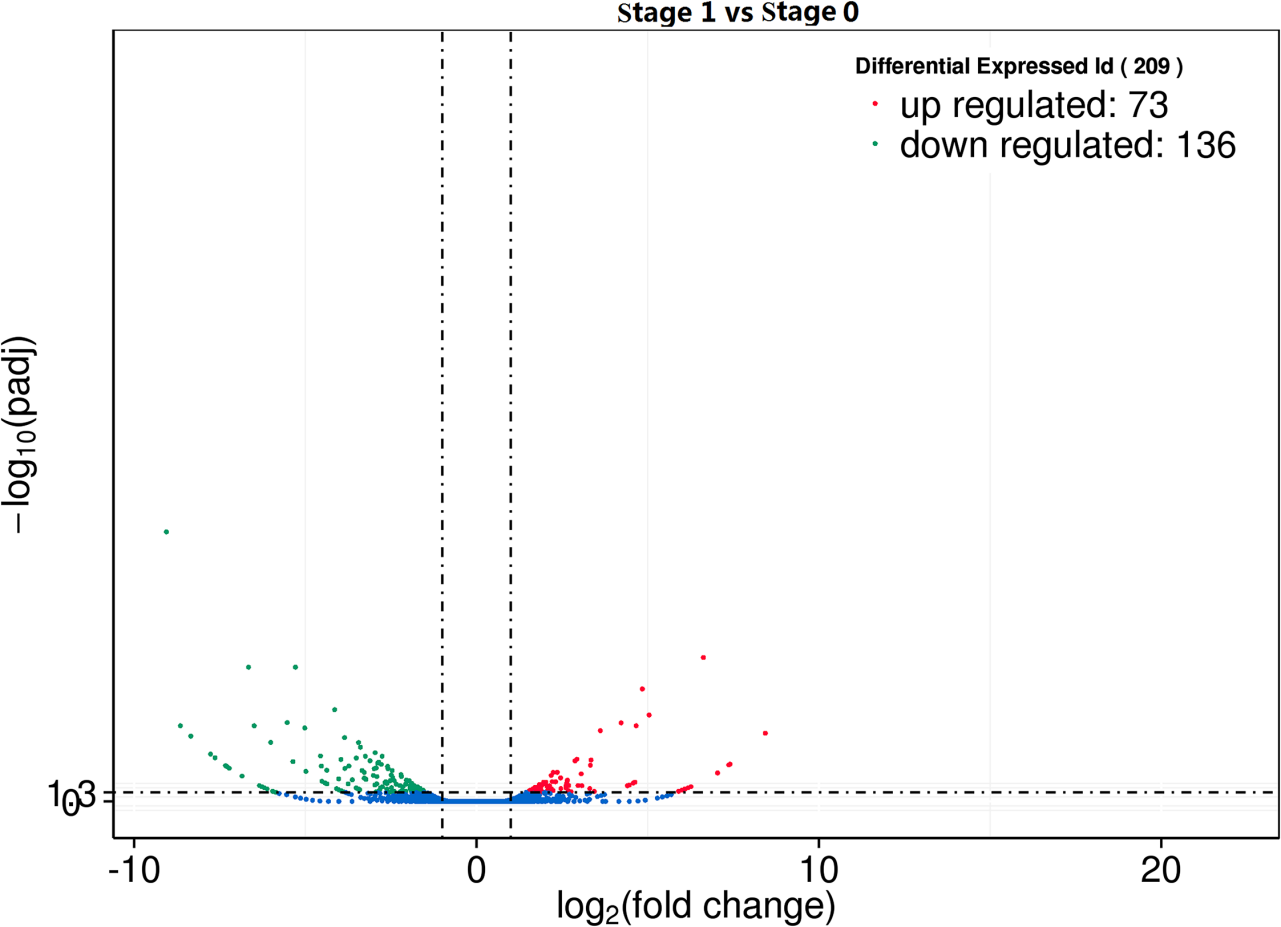
Differentially expressed genes and lncRNAs in sheep skin between stage 0 and stage 1 of secondary HF morphogenesis. Of the 209 differentially expressed genes and LncRNA, 73 were upregulated (right, red) and 136 were downregulated (left, green) in stage 1 compared with stage 0.

**Fig 6:**
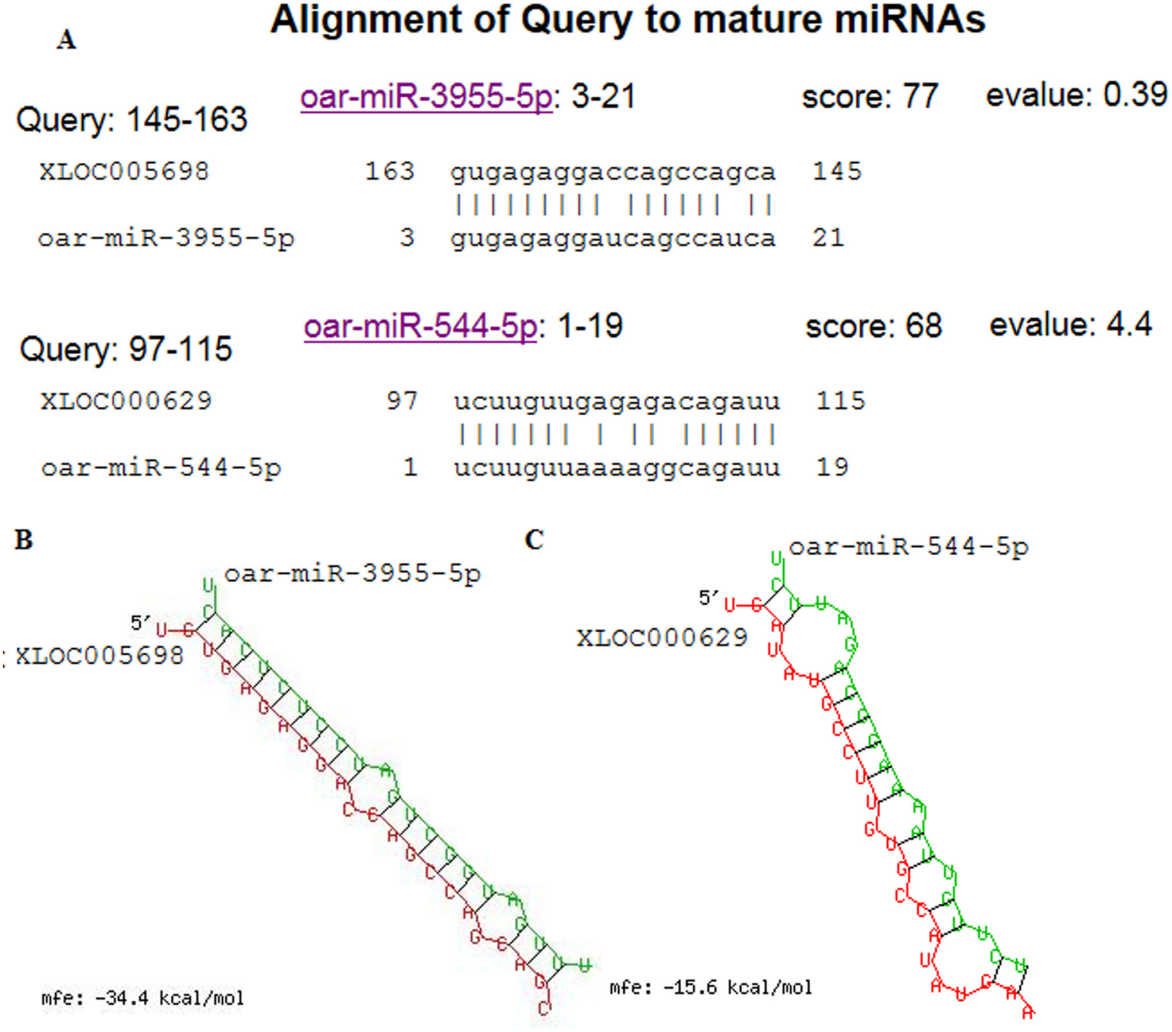
Bioinformatics predicted oar-miR-3955-5p binding sites on XLOC005698, and oar-miR-544-5p binding sites on XLOC000629. (a) Alignment between XLOC005698 and oar-miR-3955-5p as well as between XLOC000629 and oar-miR-544-5p. (b) The oar-miR-3955-5p miRNA binding sites on XLOC005698. (c)The oar-miR-544-5p miRNA binding sites on XLOC000629.

Using edgeR (the threshold is usually set as |log2 (Fold Change)| > 1 and q value < 0.005), the differential genes and lncRNAs between Stage 0 and Stage 1 group were screened, resulting in 209 differentially expressed genes and lncRNAs (Fig 5). Of these genes and lncRNAs, 67 genes and six lncRNAs were upregulated, and 127 genes and nine lncRNAs were downregulated (S1 Table). In the differentially expressed genes, the downregulated gene showing the biggest difference was *ADIPOQ* (Gene ID ENSOARG00000020509) with FPKM value of -9.058 (q value (padj) = 1.20E-39); the upregulated gene showing the biggest difference is *COL3A1* (Gene ID ENSOARG00000016476), with a multiple differential expression value of 21.90 (q value (padj) = 7.06E-107). The differentially expressed lncRNAs included 12 lincRNAs, one intronic lncRNA and one anti-sense lncRNA. Six of the differentially expressed lncRNAs were distributed on the sense strand of the sheep reference genome, and nine of these lncRNAs were distributed on the antisense strand (Table 2). XLOC_002747, XLOC_005698 and XLOC_014751 had alternative splice variants. The target gene prediction for the differential lncRNAs showed the existence of *cis* or *trans* target genes in twelve lncRNAs, but *cis or trans* target gene failed to be predicted in three lncRNAs, as XLOC007757, XLOC005698 and XLOC000629 (Table 3). Among of differentially expressed genes and LncRNA, lncRNA XLOC002437 and potential target gene *collagen type VI alpha 6* (*COL6A6*, Gene ID: 101111424) were all significantly down-regulated in stage 1 of the secondary HF morphogenesis. BLAST alignment analysis was performed for the differential lncRNAs with the mature miRNA of sheep from the miRBase database, and the results showed a high consistency between XLOC005698 and oar-miR-3955-5p as well as between XLOC000629 and oar-miR-544-5p (Fig 6A).The miRNA binding sites on LncRNAs were predicted using a web-based program RNAhybrid (version: 2.2)[29].The minimum free energy (MFE) of XLOC005698 combined with oar—miR—3955-5 p was more lower than XLOC000629 combined with oar—miR—544-5 p, the mfe was -34.4 kcal/mol(Fig 6B),-15.6 kcal/mol (Fig 6C),respectively.It was suggested that XLOC005698 may be play important roles in the regulation of gene expression during the secondary HF morphogenesis by ‘‘sponges” oar-miR-3955-5p activity as a competing endogenous RNA (ceRNA).To further obtain the potential biological functions of the differential lncRNAs, cluster analysis was performed for the differentially expressed genes and lncRNAs. The 15 differential lncRNAs were first clustered into 12 small gene clusters as follows: XLOC_000629, XLOC_002747, 101123159 and 101120453 gathered in a cluster; XLOC_021775, XLOC_006635 and 101120775 gathered in a cluster; XLOC_007281, XLOC_014751 and 101121257 gathered in a cluster, and the remaining nine lncRNAs gathered respectively into eight gene clusters (S2 Table). These results preliminarily indicated that the differential lncRNAs might be involved in secondary follicle morphogenesis and the formation of placodes through a number of different metabolic processes or cellular pathways.

**Table 2:** Differentially expressed LncRNA in sheep skin between Stage 0 and Stage 1 group of secondary HF morphogenesis.

LncRNA ID	Location	Length	Strand	Exon number	Type	Stage 1 FPKM	Stage 0 FPKM	log2Fold Change	padj
XLOC016657	chr3:214782109–147825962	219	-	2	anti-sense lncRNA	0	30.2983	-6.12044	0.015537
XLOC002437	chr1:269765163–269812147	390	-	2	lincRNA	14.6368	61.9706	-2.06719	0.003173
XLOC020709	chr9:32640662–32645592	4043	+	3	lincRNA	3.85757	0.11348	5.040358	3.36E-13
XLOC014751	chr3:15940142–15943214	479	+	2,3	lincRNA	5.92145	0.242519	4.407241	0.005568
XLOC007281	chr15:69763640–70034206	1328	-	2	lincRNA	6.15907	0.603154	3.326677	5.94E-06
XLOC007757	chr17:4435904–4441701	3610	+	2	lincRNA	3.74209	1.20535	1.63523	0.049143
XLOC005698	chr14:32625557–32704885	924	+	2,3	lincRNA	7.18229	1.24453	2.098051	0.014642
XLOC014287	chr25:33206921–33212098	5112	-	2	lincRNA	1.80369	0.431517	2.059673	0.006547
XLOC021775	chrX:94517001–94546262	4043	-	6	lincRNA	3.85757	0.11348	5.040358	3.36E-13
XLOC006635	chr14:56674446–56681460	753	-	2	lincRNA	0.730852	5.31213	-2.80751	0.010757
XLOC018246	chr5:11050873–11083559	1296	-	4	lincRNA	0.372759	5.13388	-3.72663	7.75E-06
XLOC000629	chr1:41283122–41284685	1460	+	2	IntroniclncRNA	0.27059	2.65364	-3.22868	0.000668
XLOC002747	chr10:30088157–30091646	1128	-	2,3	lincRNA	0.342813	3.02487	-2.96701	0.004374
XLOC003435	chr11:57875653–57891453	2715	+	2	lincRNA	1.22596	8.9283	-2.85311	2.21E-06
XLOC016440	chr3:171509716–171564468	2506	-	3	lincRNA	1.48499	5.01501	-1.74615	0.030153

**Table 3:** Potential *cis* or *trans* target gene,ncRNA,micRNA of differentially expressed LncRNA.

LncRNA ID	Potential target gene/ncRNA/micRNA			
	Gene ID	Gene symbol	Description	Cis/ Trans
XLOC016657	101122943	TAB1	TGF-beta activated kinase 1	Cis
	105614926	SYNGR1	synaptogyrin 1	Cis
	101106836	RPL3	ribosomal protein L3	Cis
XLOC002437	101111424	COL6A6	collagen, type VI, alpha 6	Cis
	101107574	KIAA1715	KIAA1715 ortholog	Trans
XLOC020709	101111889	EFCAB1	EF-hand calcium binding domain 1	Cis
	105616018		LOC105616018(Gene type:ncRNA)	Cis
	105609197		LOC105609197(Gene type:ncRNA)	Cis
XLOC014751	105607455		LOC105607455(Gene type:ncRNA)	Cis
	105607456		LOC105607456(Gene type:ncRNA)	Cis
XLOC007281	101108158	LRRC4C	leucine rich repeat containing 4C	Cis
	105602356		LOC105602356(Gene type:ncRNA)	Cis
	101115653		LOC101115653(Gene type: pseudogene)	Cis
XLOC005698	MIMAT0019245	-	oar-miR-3955-5p	Trans
XLOC014287	101113003	KLK12	kallikrein-related peptidase 12	Trans
	105605013		LOC105605013(Gene type:ncRNA)	Trans
XLOC021775	101107015	TMEM196	transmembrane protein 196	Trans
	105605574		LOC105605574(Gene type:ncRNA)	Cis
	105605574		LOC105605573(Gene type:ncRNA)	Cis
XLOC006635	101103089	ZNF577	zinc finger protein 577	Cis
	101104336		LOC101104336	Cis
	101104087	ZNF614	zinc finger protein 614	Cis
	101102585	HAS1	hyaluronan synthase 1	Cis
	101114548	ZNF613-like	zinc finger protein 613-like	Cis
XLOC018246	101102642	ZNF791	zinc finger protein 791	Cis
XLOC000629	MIMAT0019298		oar-miR-544-5p	Trans
XLOC002747	101110772	HSPH1	heat shock 105kDa/110kDa protein 1	Cis
	105616258		WD repeat-containing protein 49-like	Cis
XLOC003435	101107975	SOX9	SRY (sex determining region Y)-box 9	Cis
	101103746	PLAGL2	pleiomorphic adenoma gene-like 2	Trans
	101121742	RID5B AT	rich interactive domain 5B	Trans
XLOC016440	101121522	MCC	MCC mutated in colorectal cancers	Trans
	101105703	PLCE1	phospholipase C, epsilon 1	Trans

**Fig 7:**
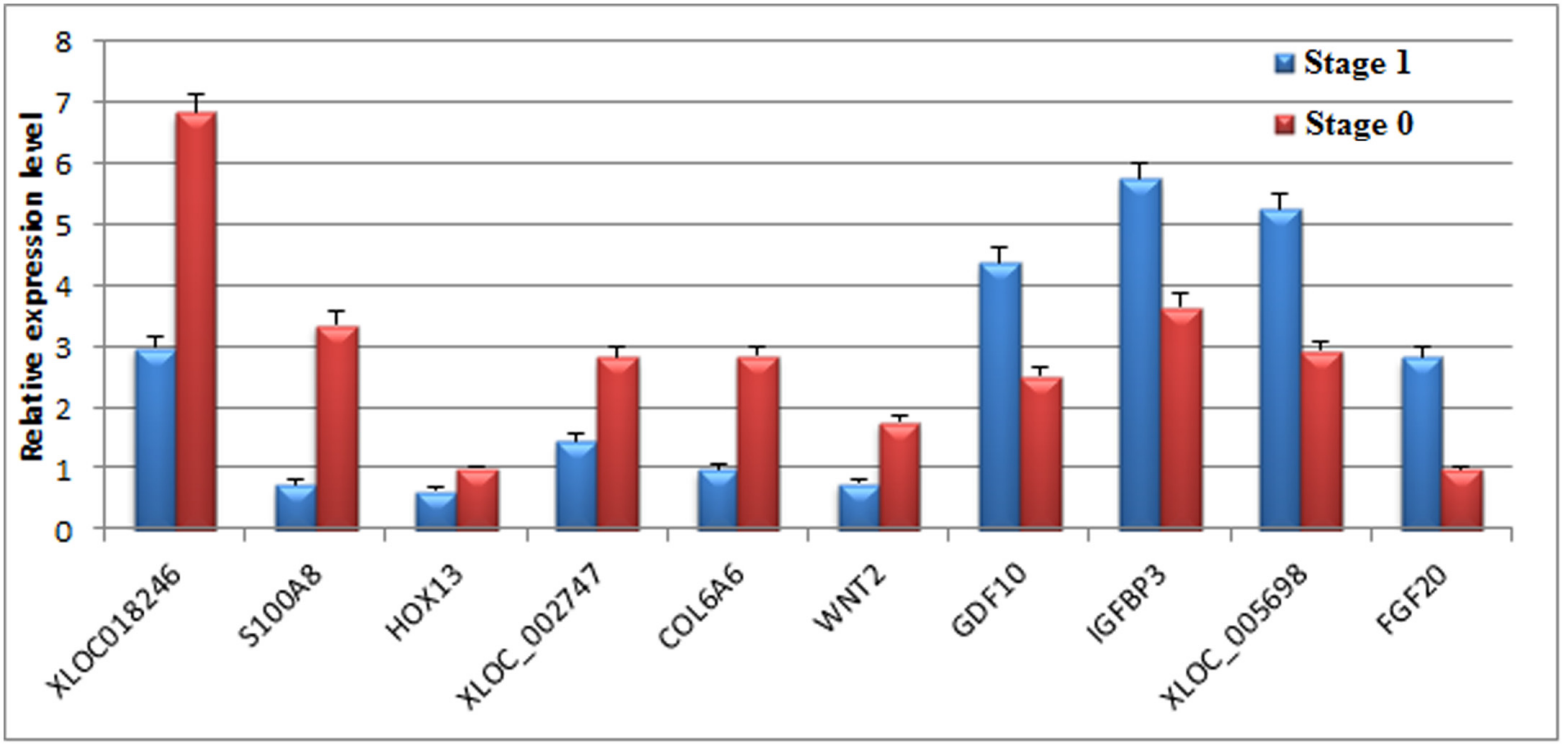
The expression level of differently expressed genes and LncRNAs validated by strand-specific qPCR.

The differentially expressed genes and lncRNAs obtained from the screening were verified by strand-specific RT-PCR. Nine differentially expressed genes and lncRNAs were randomly selected from the two groups, and *GAPDH* was used as the internal reference. The quantitative results showed that the expression patterns of the selected differentially expressed genes in the two groups were consistent withFPKM values of these genes and lncRNAs (Fig 7), and the sequencing results correlated with the strand-specific RT-PCR results.

### Enrichment of differentially expressed genes and lncRNAs

**Fig 8:**
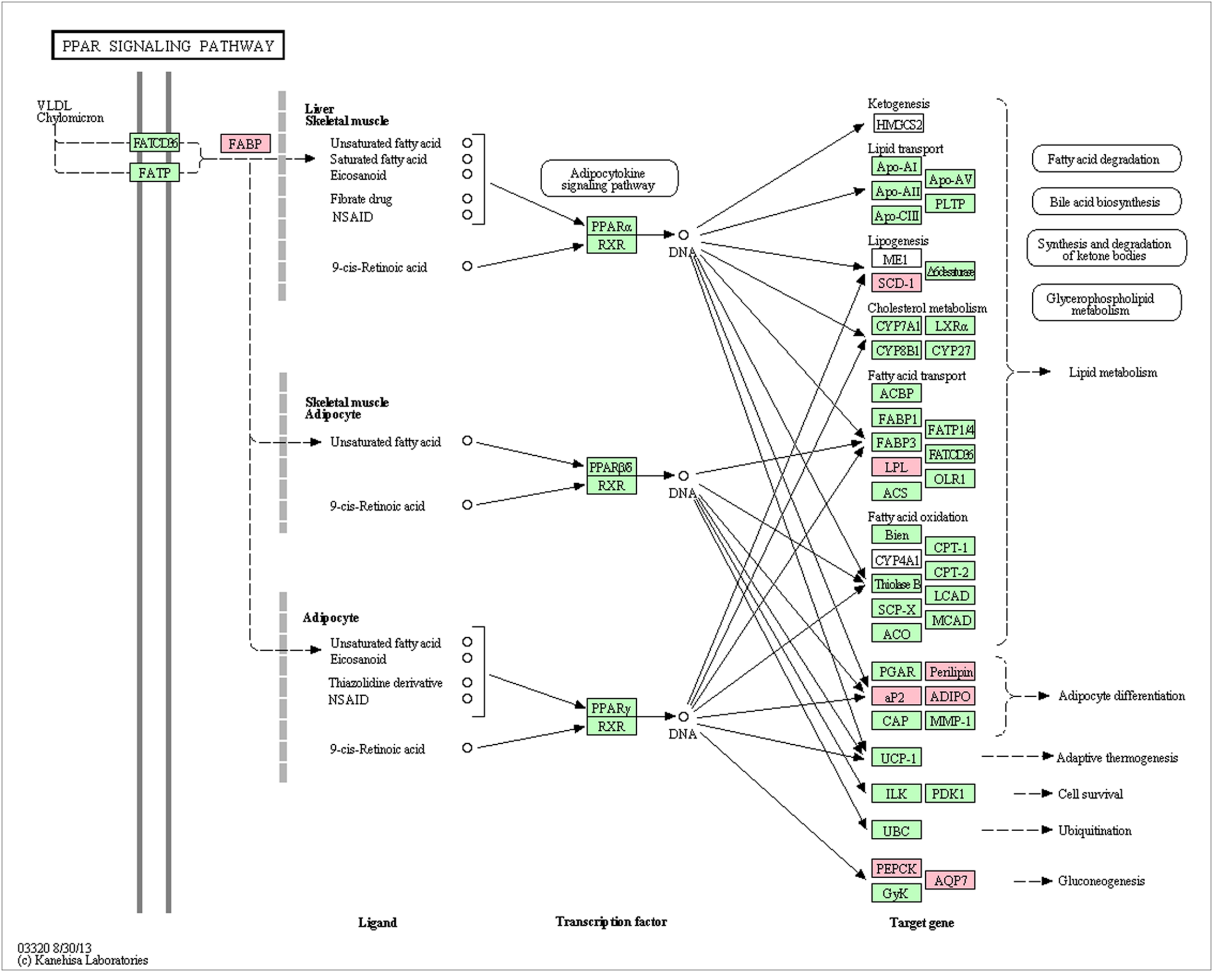
Differentially expressed genes between Stage 0 and Stage 1 of secondary HF morphogenesis involved in PPAR signaling pathway. The red color labels genes significantly down regulated in stage 1 compared with stage 0(q value < 0.005).The green color labels genes were expressed in sheep skin during secondary HF initiation, but not significant difference.

The 160 differentially expressed genes and target genes of 12 lncRNAs containing the functional annotation information were assigned to 1,023, 191GO terms,respectively. There were five, five and seven GO terms that were significantly enriched for biological process, molecular function and cellular components, respectively (corrected P-value < 0.05) (Table 4).The differential genes in the induction stage of the secondary follicle morphogenesis in sheep were enriched into 136 pathways, including the PPAR signaling pathway, ECM-receptor interaction, the PI3K-Akt signaling pathway, the Wnt signaling pathway, the VEGF signaling pathway and the MAPK signaling pathway. Of these pathways, 8 genes of the PPAR signaling pathway was significantly enriched (corrected P-value < 0.05) (Fig 8, S3 Table).

**Table 4:** Gene ontology analysis of differentially expressed genes in sheep skin between Stage 0 and Stage 1of secondary HF morphogenesis.

GO_accession	Description	Term type	Corrected pValue
GO:0046849	bone remodeling	biological process	1.13E-05
GO:0048771	tissue remodeling	biological_process	1.13E-05
GO:0044707	single-multicellular organism process	biological_process	0.032134
GO:0032501	multicellular organismal process	biological_process	0.032863
GO:0006952	defense response	biological_process	0.046941
GO:0005576	extracellular region	cellular_component	2.44E-05
GO:0044421	extracellular region part	cellular_component	0.0006
GO:0005615	extracellular space	cellular_component	0.001997
GO:0005882	intermediate filament	cellular_component	0.044882
GO:0045111	intermediate filament cytoskeleton	cellular_component	0.044882
GO:0004857	enzyme inhibitor activity	molecular_function	2.44E-05
GO:0030414	peptidase inhibitor activity	molecular_function	2.44E-05
GO:0061134	peptidase regulator activity	molecular_function	2.44E-05
GO:0004866	endopeptidase inhibitor activity	molecular_function	2.44E-05
GO:0061135	endopeptidase regulator activity	molecular_function	2.44E-05
GO:0004869	cysteine-type endopeptidase inhibitor activity	molecular_function	0.00072
GO:0030234	enzyme regulator activity	molecular_function	0.017937

## Discussion

Initiation of HF involves a series of signaling between the epidermal cell and the dermal papilla, such as Wnt/beta-catenin, EDA/EDAR/NF-κB, Noggin/Lef-1, Ctgf/Ccn2, Shh, BMP-2/4/7, Dkk1/Dkk4 and EGF [30, 31]. This study also found that the above signaling molecules were expressed in sheep skin during the initiation of secondary HF. However,the astonishing thing is that only *Wnt2*, *FGF20* were just significant differentially expressed. In this study, we observed that *Wnt2* at stage 1 is 1.58 times less expressed than at stage 0 in sheep skin. Cadau *et al* also observed a maximal expression of *Wnt2* at E12.5 in mouse skin and a slight decrease at E13. This decrease is more significant at E13.5, reaching a third of its initial expression. At E14.5, *Wnt2* is ten times less expressed than at E12.5 [14]. *In situ hybridization* has revealed that *Wnt2* is expressed in the epidermis and HF during the HF placode stage [32]. Therefore, *Wnt2* likely acts as the secondary Wnt[32], which is a part of the placode signal and maybe is very essential for sheep HF initiation. Immediately after the formation of the placode, the dermal fibroblasts are aggregated, which is regulated by *fibroblast growth factor 20* (*FGF20*), which is induced by epithelial *Eda/Edar* and *Wnt/β-catenin* and expressed in the HF placode [33]. *FGF20* controls the aggregation of primary and secondary follicle dermis. *FGF20* is also significantly upregulated at stage 1 of secondary HF, while *FGFR1*, *FGFR2*, *FGFR3* and *FGFR4*, as potential *FGF20* receptors, are not significant[33].

Recent studies have shown that the morphogenesis of HFs is not only related to the proliferation and differentiation of HF cells but also affected by other cells around the HFs,such as sebaceous gland, sweat gland[34–36]. Peroxisome proliferator-activated receptors (PPARs) are members of the nuclear hormone receptor family and have emerged as the important mediators of lipid metabolism in adipocytes and sebaceous glands [37, 38]. A set of different *in vitro* studies has demonstrated the important function of PPARs for cell differentiation, lipid synthesis and fatty acid uptake into cells [38]. Additionally, *SCD1* and *PPARs* have also been implicated in the regulation of keratinocyte differentiation and the formation of a functional skin barrier [39, 40]. *FATP4*^*-/-*^, *Dgat1*^*-/-*^, *Dgat2*^*-/-*^ and Early B cell factor 1 (*Ebf1*^*-/-*^) mice have decreased intradermal adipose tissue due to defects in lipid accumulation in mature adipocytes [41–43]. Interestingly, these mice also display abnormalities in skin structure and function such as hair loss and epidermal hyperplasia. The results of this study showed that the PPAR signaling pathway was significantly enriched (corrected P-value < 0.05) and *FABP4*, *AQP7*, *ADIPOQ*, *PEPCK*, *SCD*, *LPL* and *PLIN1* in PPAR signaling pathway were significantly downregulated at stage 1 of secondary HF. A previous study revealed that sebaceous gland buds form on the ental side of central primary HF during secondary HF morphogenesis on day 90 [44]. It is speculated that secondary HF morphogenesis in sheep may be promoted by reducing PPAR and then inhibiting the formation of sebaceous glands around the primary HFs.

LncRNA is a class of RNA molecules that do not encode proteins, and lncRNAs are 200 bp or more in length and have conserved secondary structures. LncRNAs may interact with proteins, DNA and RNA, and their biological function is involved in a variety of mechanisms at multiple levels of epigenetic, transcriptional and post-transcriptional levels. These mechanisms include gene imprinting, chromatin remodeling, cell cycle regulation, splicing regulation, mRNA degradation and translational regulation [45]. The databases of NONCODE mainly include human and mouse lncRNA data. In the NONCODE v4.0 and lncRNAdb database, lncRNAs have been rapidly increased from 73,327 to 210,831 in the last two years, respectively. This is indicated that lncRNAs are becoming a hot topic in life science research. However, only five sheep lncRNAs (*antiPeg11* [46], *MEG3*, *MEG9*, *Rian and Xist* [47]) can be found in the above database [48]. This study obtained 884 novel sheep lncRNAs. A total of 15 lncRNAs with significant differential expression were detected, 6 up-regulated and 9 down-regulated. The *cis* or *trans* target gene prediction for lncRNAs showed LncRNA, XLOC002437 and potential target gene COL6A6 were all significantly expression. *COL6A6* is expressed in a wide range of fetal and adult tissues including lung, kidney, liver, spleen, thymus, heart, skeletal muscle and skin dermis [49, 50]. Thus, defective *COL6A6* results in the disorders with combined muscle and connective tissue involvement, including weakness, joint laxity and contractures, as well as abnormal skin [51–53]. The expression of collagen VI chains is highly regulated at different levels, such as gene transcription, processing of encoding RNAs, translation and post-translational modifications, and the impairment of the efficiency of each step may affect protein assembly and secretion [53, 54]. The XLOC002437 lncRNA and COL6A6 are significantly downregulated at stage 1 of secondary HF, suggesting that the interaction of COL6A6 and XLOC002437 may regulate and reduce the collagen VI α6 chain deposition in the skin by positive feedback, thereby inhibiting skin fibrosis and promoting the formation and deposition of the placode.

Studies have shown the relationship of mutual regulation among miRNAs, lncRNAs and mRNAs [55–58]. Recent studies show that lncRNA can interact with the miRNA as a competing endogenous RNA(ceRNA) to participate in the expression regulation of target genes, which exert an important role in the initiation and progression of tumor[59, 60]. For example,the LncRNA H19 as a ceRNA for miR-138 and miR-200a promotes epithelial to mesenchymal transition by functioning as miRNA sponges in colorectal cancer[59]. In this study, we found the high consistency between XLOC005698 and oar-miR-3955-5p as well as more lower MFE. XLOC005698 may be as a competing endogenous RNA ‘‘sponges” oar-miR-3955-5p activity. However, the molecular mechanism of the interaction of oar-miR-3955-5p and XLOC005698 lncRNA also need further study.

## Conclusions

The present study applied the ssRNA-seq technique to integrated analysis of the role of LncRNA and coding RNA expression in sheep (*Ovis aries*) skin during the initiation of secondary HF.A total of 884 novel lncRNAs were discovered in sheep skin expression profiles. Differences were found in 192 expressed genes, 15 lncRNAs between the two different stages.These results laid a foundation to screen the regulatory elements or functional genes that specifically regulate the initiation of secondary HF, as well as supplying some potential values for understanding human hair disorders.

## Materials and Methods

### Sheep skin sampling

Alpine merino sheep were obtained from a sheep stud farm located in Zhangye City, Gansu Province. All experimental and surgical procedures were approved by the Biological Studies Animal Care and Use Committee, Gansu Province, People’s Republic of China. Sixty GAS ewes (2–3 years old), which had a mean fiber diameter of 18.1±0.5 μm and were sourced from a single flock, were artificially inseminated with fresh sperm from a single ram (fiber diameter = 19.20 μm), and the day of insemination was designated as embryonic day (E) 0. Three fetuses were collected at E87(stage 0 of HF morphogenesis) and E96(stage 1 of HF morphogenesis), respectively[61]. On the day of sampling, the pregnant ewes were stunned via captive bolt and exsanguinated. The uterus was exteriorized, and the fetuses were carefully removed. The fetuses were washed in phosphate buffered saline and exsanguinated. The midside skin strips from two sides of the fetuses were snap frozen in liquid nitrogen for frozen sectioning and RNA extraction.

### HF morphogenesis

Frozen skin strips from E87 and E96 were embedded in an O.C.T. compound (Sakura Finetek, USA, Inc., Torrance, CA), cut into 8-μm-thick serial sections in a cryostat, placed on Superfrost Plus glass slides (Fisher Scientific, Pittsburgh, PA, USA) and stained with hematoxylin. HF morphogenesis was studied in the longitudinal direction serial sections to observe secondary follicle morphogenesis.

### Total RNA extraction, library construction and deep sequencing

Total RNA was isolated from the tissues using an RNeasy Maxi Kit (Qiagen, Hilden, Germany) according to the manufacturer’s instructions. RNA quality was verified using a 2100 Bioanalyzer RNA Nano Chip (Agilent, Santa Clara, CA, USA), and the RNA Integrity Number (RIN) value was > 8.5. The RNA was quantified using a Nano Drop ND-2000 Spectrophotometer (Nano-Drop, Wilmington, DE, USA).

The rRNA was depleted from 3 μg of total RNA using Epicentre Ribo-Zero™ rRNA Removal Kit (Epicentre, USA). The cDNA libraries were prepared from the remaining RNA without poly(A) selection using the NEBNext^®^ Ultra^™^ Directional RNA Library Prep Kit for Illumina (NEB, USA) according to the manufacturer’s instructions. The products were then purified with AMPure XP Universal PCR primers and the Index (X) Primer. The products were purified (AMPure XP system), and library quality was assessed using the Agilent Bioanalyzer 2100 system. The clustering of the index-coded samples was performed on a cBot Cluster Generation System using the TruSeq PE Cluster Kit v3-cBot-HS (Illumina). After cluster generation, the libraries were sequenced on the Illumina HiSeq 2000 platform, and 125 bp paired-end reads were generated.

Sequencing-received raw image data were transformed by base culling into sequence data, which was called raw data. Raw sequences were transformed into clean reads after removing all low quality tags, empty reads and singletons (tags that occurred only once). All paired-end clean reads were mapped to sheep reference sequences (version:Oarv3.1) by TopHat2 (version:V2.0.9) [25], and read counts for different genes and other known transcripts (misc_RNA, pseudogene, rRNA, tRNA and others) were extracted by HTSeq (version: V0.6.1) [62] with default parameters and allowing one mismatch. To monitor mapping events on both strands, both sense and complementary antisense sequences were included [63].

### Identification of lncRNAs

LncRNAs were identified using the following workflow. According to the annotation of sheep reference sequences (version:Oarv3.1), the transcriptome from each dataset was assembled independently using the Cufflinks package (version:2.1.1) [26] and Scripture (version:beta2) [27]. Transcripts smaller than 200 bp or all single-exon transcripts were excluded first. Cufflinks was then used to estimate the abundance of all transcripts based on the final transcriptome, and the transcripts with coverage less than 3 were also discarded. All transcriptomes were then pooled and merged to generate a final transcriptome using Cuffmerge, and the known protein-coding transcripts as well as rRNA, tRNA, snRNA, snoRNA, pre-miRNA and pseudogenes were identified using Cuffcompare and excluded. The remaining unknown transcripts were used to screen for putative lncRNAs. Among the different classes of class_code (http://cufflinks.cbcb.umd.edu/manual.html#class_codes), only those annotated by “u”, “i”, and “x” were retained, which represent potential novel intergenic, intronic and anti-sense lncRNAs, respectively. Filtering of the remaining transcripts resulted in many novel, long expressed transcripts. We first used CNCI [64] and PhyloCSF [65] to predict transcripts with coding potential. All transcripts with CNCI scores > 0, CNCI scores > 0 or PhyloCSF score > 100 were discarded. The remaining transcripts were subjected to HMMER analysis to exclude transcripts that contained any known protein domains cataloged in the Pfam database[66]. Conservation analysis of lncRNAs and mRNAs was performed using phastCons with default parameters [67]. To select bona fide lncRNAs, the lncRNAs identified using the above four methods were integrated into a comprehensive data set.

### Determination of gene expression levels and detection of DEGs and lncRNAs

Gene expression FPKM values of mRNAs and lncRNAs were calculated with Cufflinks v2.0.2. Additionally, a table comprising read counts for each transcript was calculated using BEDTools version 2.17.0 [68]. We removed the low expressed transcripts (at least all of the samples had FPKM < 0.1). The set of remaining transcripts was reduced to a set of non-overlapping regions (or 'genes') by comparing all overlapping transcripts and keeping the transcript with the largest average FPKM across all samples as the representative transcript for that region. For differential expression quantification of mRNA and lncRNA genes, EdgeR version 3.0.8 [69] was used to identify differentially expressed transcripts between E87 and E96 using q value (p-adjusted) ≤ 0.05 and absolute fold change ≥ 1.

Differentially expressed lncRNAs were selected for target prediction via *cis-* or *trans*-regulatory effects. For the cis pathway target gene prediction, lncRNAs and potential target genes were paired and visualized using UCSC genome browser on the NCBI database. The genes transcribed within a 100 kbp window upstream or downstream of lncRNAs were considered as *cis* target genes [70]. For the *trans* pathway target gene prediction, the blast ratio (e < 1E-5) between lncRNAs and protein coding genes was calculated. RNAplex software was then used to select *trans*-acting target genes [71]. RNAplex parameters were set as -e -20. For the prediction of target miRNAs, lncRNAs were screened in the sense-antisense miRNA overlapping and non-overlapping regions by searching for similarity with miRBase mature miRNA sequences of sheep (*Ovis aries*) [72] using the BLAST program. The miRNA-binding sites on lncRNAs were then predicted using a web-based program called RNA hybrid (version: 2.2) [29].

### Strand-specific real-time quantitative RT-PCR

To confirm the differentially expressed sense and antisense transcripts between super fine wool group and fine wool group, ten genes were randomly selected to verify the expression levels of genes and LncRNAs in skin by strand-specific qRT-PCR according to the protocol described in yue *et al*. (2015)[19]. Primers for real-time PCR were designed with Primer Express 3.0 (Applied Biosystems) (S4 Table).

### GO and KEGG enrichment analysis of differentially expressed genes and LncRNA

All differentially expressed genes and the predicted target genes of the LncRNAs were mapped to GO terms in the GO database, and the gene numbers for each GO term were calculated using the GO seq R package (version:1.18.0) [73]. The significantly enriched metabolic pathways or signal transduction pathways were identified via pathway enrichment analysis using KEGG(Kyoto Encyclopedia of Genes and Genomes), and KOBAS (version: 2.0) [74].In above all tests, P-values were calculated using Benjamini-corrected modified Fisher´s exact test, and ≤ 0.05 was taken as a threshold of significance GO terms or pathways.

## Supporting Information

S1 Fig Comparison of transcript length,ORF length, the number of exons and conservation score of mRNA and LncRNA. (A) The transcript length distribution of mRNA and LncRNA.(B)The ORF length distribution of mRNA and LncRNA. (C) the number of exons of mRNA and LncRNA.(D) The cumulative distribution curve of mRNA and LncRNA conservation scores.
(PDF)

S1 Table Differentially expressed genes and LncRNA between Stage 0 and Stage 1 of secondary HF initiation. (XLS)

S2 Table Cluster genes of differentially expressed LncRNAs in sheep skin during secondary HF initiation. (XLS)

S3 Table Differentially expressed genes of PPAR signaling pathway in sheep skin between Stage 0 and Stage 1 of secondary HF initiation. (XLS)

S4 Table Relevant information of gene and primer sequences for strand-specific RT-PCR. (XLS)
