# Supplementary figures and images for: Integrated Analysis of the Roles of Long Noncoding RNA and Coding RNA Expression in Sheep (Ovis aries) Skin during Initiation of Secondary Hair Follicle
Source: PLoS One. 2016 Jun 8;11(6):e0156890. doi: 10.1371/journal.pone.0156890 (PMC4898689; doi:10.1371/journal.pone.0156890)

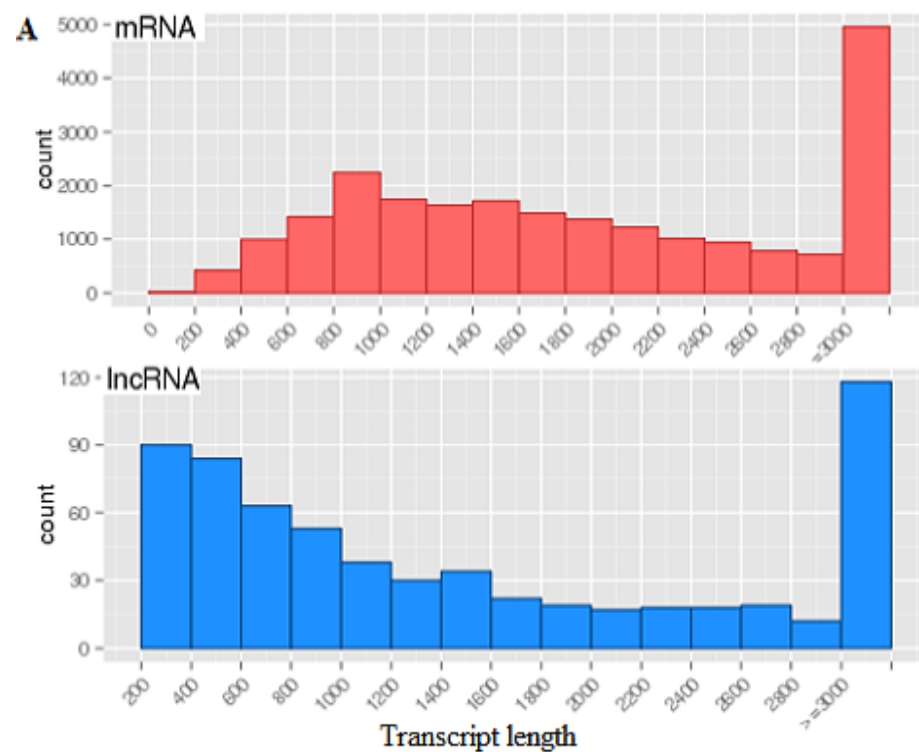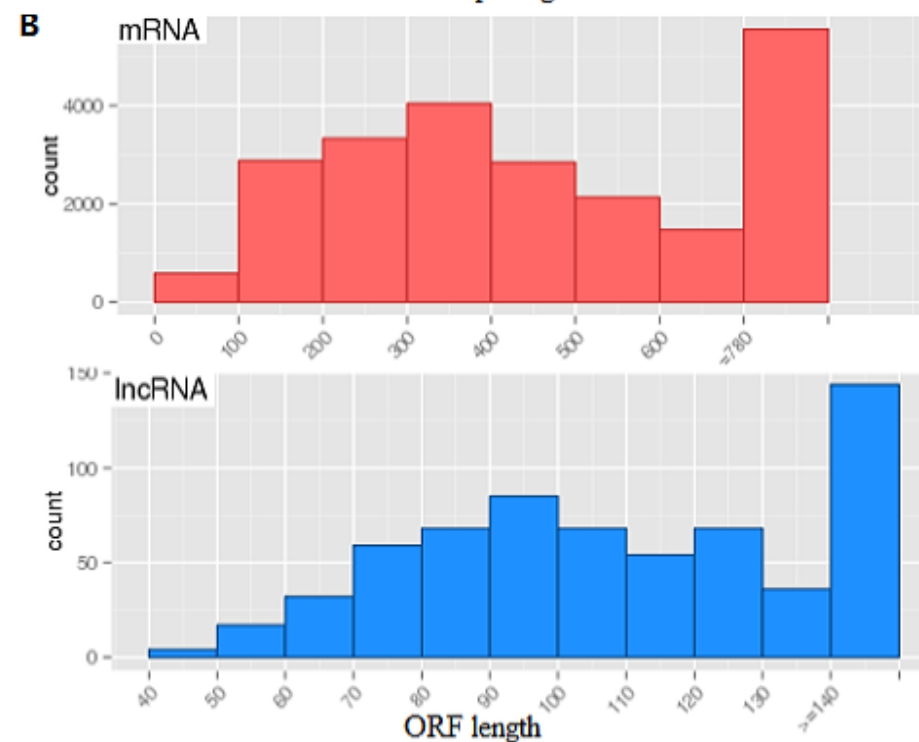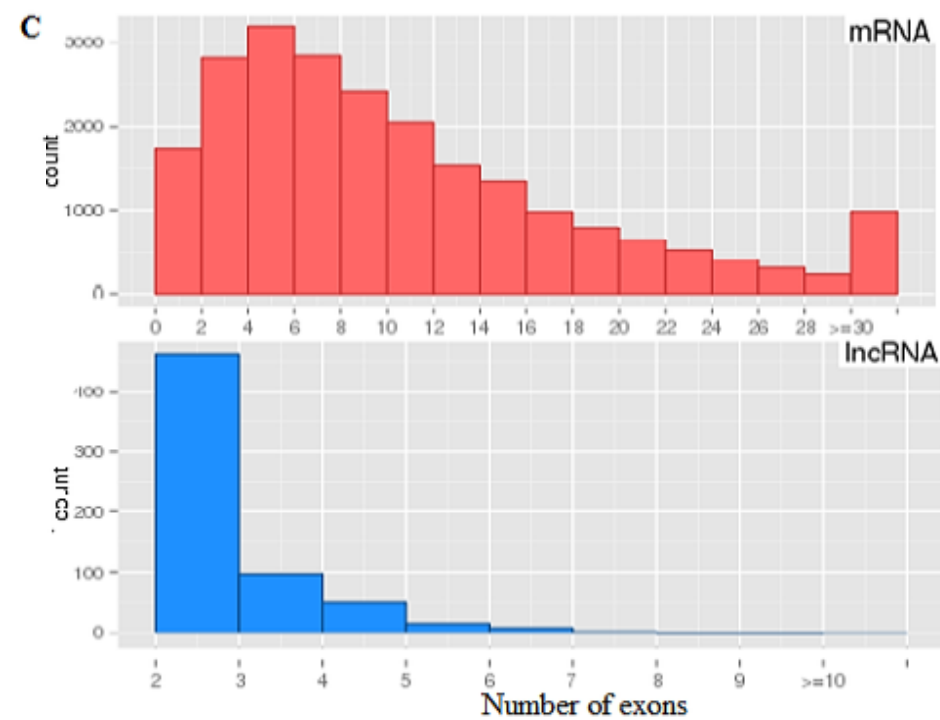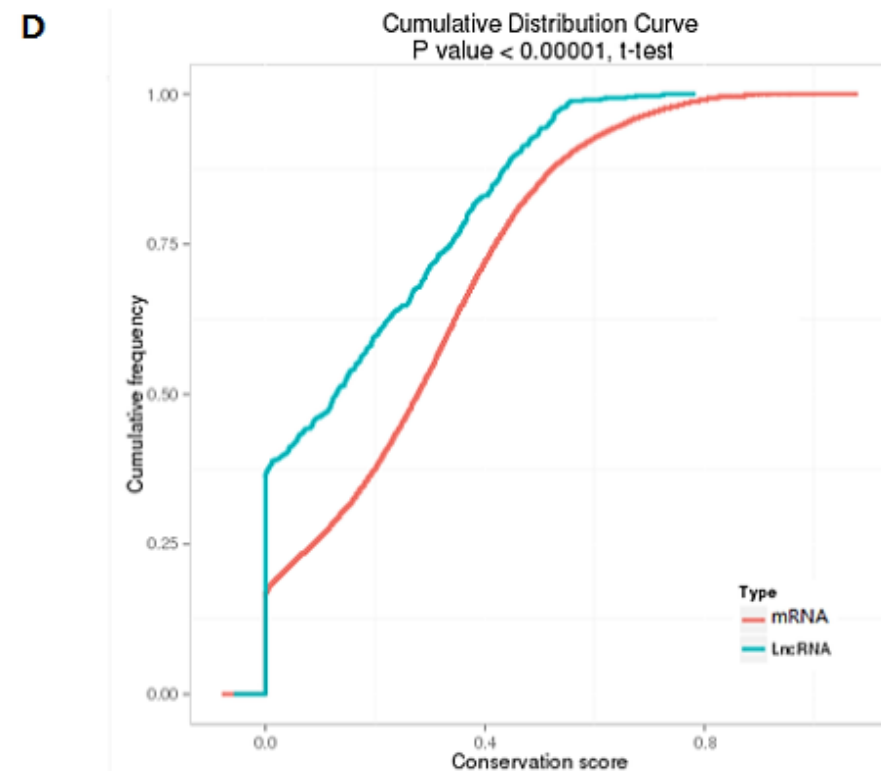

Supplement: S1 Fig — (A) The transcript length distribution of mRNA and LncRNA.(B)The ORF length distribution of mRNA and LncRNA. (C) the number of exons of mRNA and LncRNA.(D) The cumulative distribution curve of mRNA and LncRNA conservation scores. (PDF) [file pone.0156890.s001.pdf]
